# Intrinsic cardiac ganglia and acetylcholine are important in the mechanism of ischaemic preconditioning

**DOI:** 10.1007/s00395-017-0601-x

**Published:** 2017-01-13

**Authors:** J. M. J. Pickard, N. Burke, S. M. Davidson, D. M. Yellon

**Affiliations:** The Hatter Cardiovascular Institute, University College London, 67 Chenies Mews, London, WC1E 6HX UK

**Keywords:** Myocardial infarction, Ischaemic preconditioning, Intrinsic cardiac nervous system

## Abstract

This study aimed to investigate the role of the intrinsic cardiac nervous system in the mechanism of classical myocardial ischaemic preconditioning (IPC). Isolated perfused rat hearts were subjected to 35-min regional ischaemia and 60-min reperfusion. IPC was induced as three cycles of 5-min global ischaemia–reperfusion, and provided significant reduction in infarct size (IS/AAR = 14 ± 2% vs control IS/AAR = 48 ± 3%, *p* < 0.05). Treatment with the ganglionic antagonist, hexamethonium (50 μM), blocked IPC protection (IS/AAR = 37 ± 7%, *p* < 0.05 vs IPC). Moreover, the muscarinic antagonist, atropine (100 nM), also abrogated IPC-mediated protection (IS/AAR = 40 ± 3%, *p* < 0.05 vs IPC). This indicates that intrinsic cardiac ganglia remain intact in the Langendorff preparation and are important in the mechanism of IPC. In a second group of experiments, coronary effluent collected following IPC, from ex vivo perfused rat hearts, provided significant cardioprotection when perfused through a naïve isolated rat heart prior to induction of regional ischaemia–reperfusion injury (IRI) (IS/ARR = 19 ± 2, *p* < 0.05 vs control effluent). This protection was also abrogated by treating the naïve heart with hexamethonium, indicating the humoral trigger of IPC induces protection via an intrinsic neuronal mechanism (IS/AAR = 46 ± 5%, *p* < 0.05 vs IPC effluent). In addition, a large release in ACh was observed in coronary effluent was observed following IPC (IPC_eff_ = 0.36 ± 0.03 μM vs *C*_eff_ = 0.04 ± 0.04 μM, *n* = 4, *p* < 0.001). Interestingly, however, IPC effluent was not able to significantly protect isolated cardiomyocytes from simulated ischaemia–reperfusion injury (cell death = 45 ± 6%, *p* = 0.09 vs control effluent). In conclusion, IPC involves activation of the intrinsic cardiac nervous system, leading to release of ACh in the ventricles and induction of protection via activation of muscarinic receptors.

## Introduction

Ischaemic preconditioning (IPC) is a powerful cardioprotective phenomenon, whereby brief cycles of ischaemia to a coronary bed renders it less susceptible to subsequent ischaemia-and-reperfusion-mediated infarction [31, 51]. Indeed, IPC has emerged as a highly conserved cardioprotective intervention, effective in many mammalian species via a similar mechanistic pathway (see meta-analysis [67] and recent review [29]). Although studies have reported protection by classical IPC in the setting of cardiac surgery [32], it is not practical to be used clinically in either this setting or indeed in the in the setting of acute myocardial infarction (AMI). However, the potency of cardioprotection offered by classical IPC provides a very useful tool to better understand the physiological basis of cardioprotection.

There are three interesting mechanistic traits of classical IPC: (1) there are several triggers which initiate the protective reflex; (2) a threshold exists that must be surpassed in order for protection to occur and (3) the presence of an effector signalling pathway within the cardiomyocyte is necessary. The triggers for IPC are several small molecules, released in the heart following the brief cycles of ischaemia. These include adenosine [45], opioids [14, 60, 61] and bradykinin [66]. Blocking the receptor for one of these molecules abrogates IPC; however, this can be overcome by additional cycles of brief ischaemia [21]. Thus, IPC involves release of multiple trigger molecules that, via receptor activation, converge on a common must be reached to induce cardioprotection, relating to the strength of the IPC stimulus. Protection is observed after a single ischaemic episode of 2.5 min, but not after shorter periods [42]. Moreover, the strength of protection seems to increase with the number of cycles of IPC, such that three cycles of 5-min ischaemia affords greater protection than one [67]. Unsurprisingly, very long IPC ischaemic cycles no longer provide any cardioprotection [67]. Finally, the effector pathway, which involves activation of several well-characterised pro-survival signalling pathways within the cardiomyocyte [27, 28] and renders the cell resistant to death. Perhaps the most interesting aspect of IPC is that, despite application of the intervention prior to the index ischaemia, the majority of protection is provided against reperfusion injury. Indeed, we have demonstrated that inhibition of the RISK pathway at the point of reperfusion abrogated IPC-mediated cardioprotection [26]. This necessitates a memory phase, during which the myocardium “remembers” the protective intervention prior to its employment at reperfusion. Indeed, the initial window of protection offered by IPC lasts up to 2 h prior to the index ischaemia [41, 44]. The mechanism of this apparent memory phase is as yet unclear.

Neural control of the heart is typically thought to be mediated by regions in the brainstem and spinal cord. Indeed, the autonomic ganglia that reside within the thorax and myocardium have long been thought of as monosynaptic relay stations, which serve to confer the complex processing and efferent output of the central nervous system. In fact, there exists a complex hierarchy of cardiac neural control, with sensory afferent nerves of cardiac origin found not just in central nervous system (CNS) ganglia, but also intrathoracic and intracardiac ganglia [2, 22]. Intrinsic cardiac ganglia are thus able to process sensory information and control efferent post-ganglionic autonomic firing within the heart, in the absence of any central modulation [1]. Moreover, a recent study revealed a heterogeneous population of intrinsic cardiac nerves, in particular local circuit neurons, which respond to a variety of stimuli and can influence cardiac function on a beat-to-beat basis without CNS influence [7]. Thus, complex neural processing occurs within the heart, not just in response to central efferent input, but also sensory afferent information from the myocardium. Whether these reflexes remain intact in the Langendorff isolated heart preparation, however, is yet to be investigated.

The massive sensory and ischaemic trauma associated with myocardial infarction (MI) induces dynamic morphological and phenotypic remodelling of the intrinsic cardiac nervous system, not limited to the infarcted region [25]. A ‘neural sensory border zone’ of infarction appears, with those afferents within the infarcted region becoming less sensitive, and those in the border and remote regions preserved or enhanced [57]. The influence of this neural remodelling is not yet clear, although it is thought to contribute to ventricular arrhythmogenesis [13]. In addition, these effects occur over a period of weeks following infarction, thus the acute influence of the intrinsic cardiac nervous system on IRI is yet to be understood.

The intrinsic cardiac nervous system has recently been implicated in the cardioprotection induced by remote ischaemic conditioning and vagus nerve stimulation [8, 55]. Here, we present two separate studies designed to investigate the importance of intrinsic cardiac ganglia in classical IPC; the first using an isolated perfused heart preparation, the second using a model of IPC via coronary effluent transfer.

## Materials and methods

### Materials

Dose justification was given in detail for hexamethonium and atropine in a recent publication from the same authors [55]. Briefly, hexamethonium (Sigma-Aldrich, Missouri, USA) was employed as a neuronal nicotinic acetylcholine receptor (nAChR) antagonist, at 50 µM, to achieve specificity at nAChRs within cardiac ganglia. Atropine, a muscarinic acetylcholine receptor (mAChR) antagonist, was used at a dose of 100 nM based on its affinity to the receptor (*K*_d_ = 0.36 nM).

### Animals

All animals received humane care in accordance with the United Kingdom (Scientific Procedures) Act of 1986. Male Sprague–Dawley (SD) rats were bred at a central animal unit in University College London and were used at a weight of 250–300 g throughout the study.

### Langendorff perfused heart preparation

Rats were anaesthetised with an upper left quadrant intraperitoneal injection of sodium pentobarbitone (60 mg/kg) (Animalcare, York, UK). Hearts were quickly excised via a clamshell thoracotomy and the aorta cannulated on a Langendorff apparatus to allow for retrograde perfusion of modified Krebs–Henseleit buffer (118 mM NaCl, 25 mM NaHCO_3_, 11 mM d-glucose, 4.7 mM KCl, 1.22 mM MgSO_4_·7H_2_O, 1.21 mM KH_2_PO_4_ and 1.84 mM CaCl_2_·2H_2_O. The buffer was warmed to 37.5 °C and gassed with 95% O_2_/5% CO_2_ to obtain a pH of 7.35–7.45) (for detailed methods see [9]). A fluid-filled latex balloon was inserted into the left ventricle to allow for measurement of functional parameters, including heart rate (HR) and left ventricular developed pressure (LVEDP). Coronary flow rate (CFR) was recorded throughout the protocol and the temperature of the heart was maintained at 37.0 ± 0.5°C. Finally, a 3-0 Mersilk suture (Ethicon, Edinburgh, UK) was inserted through the heart to surround the left anterior descending (LAD) coronary artery. All hearts received a 35-min LAD ischaemia and 60-min reperfusion.

## Study 1

### Classical ischaemic preconditioning

**Fig. 1:**
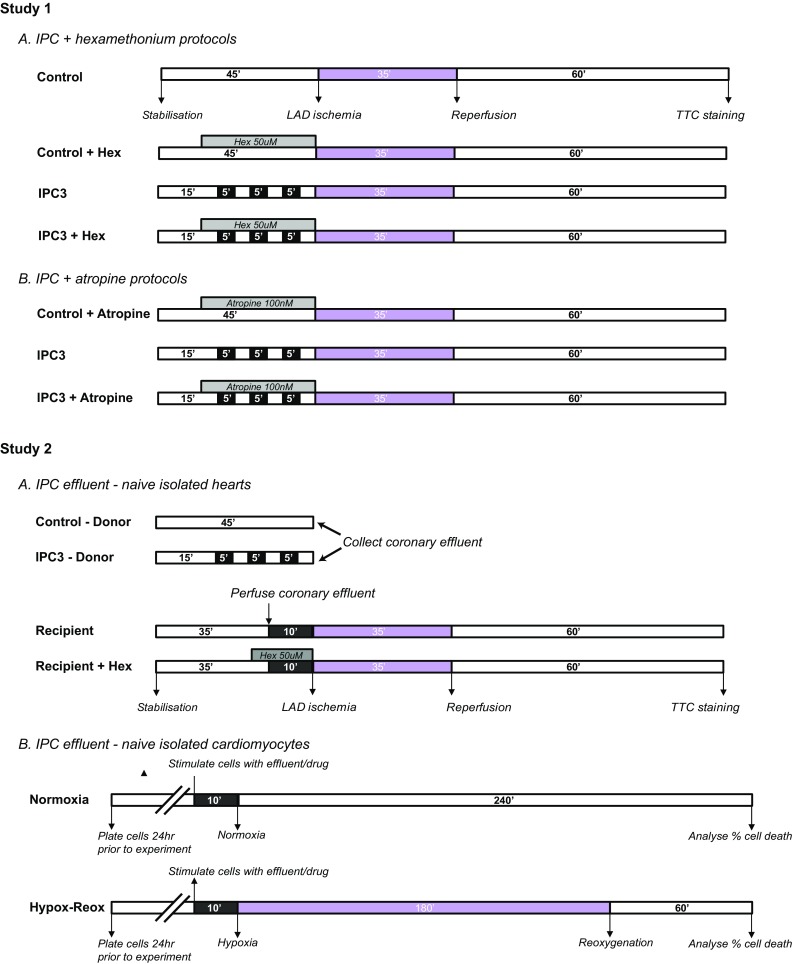
Schema detailing the experimental protocols: rat hearts were subjected to 35-min LAD ischaemia and 60-min reperfusion. Preconditioning was induced by three cycles of 5-min global ischaemia–reperfusion. Study 1, hexamethonium (*1a*) and atropine (*1b*) were perfused through the heart for 5 min prior to and for the duration of the conditioning protocol. Study 2, coronary effluent was collected from isolated hearts either following IPC or control, and subsequently perfused through (*2a*) a naïve isolated heart and prior to index ischaemia. Again, hexamethonium was perfused through the recipient heart for 5 min prior to and the duration of effluent perfusion. Coronary effluent was used to stimulate isolated cardiomyocytes (*2b*) prior to hypoxia-reoxygenation injury

Two separate experiments were designed to investigate intrinsic cardiac nerves in classical ischaemic conditioning (Fig. 1). The first experiment tested the involvement of intrinsic cardiac ganglia in IPC, via use of the nicotinic acetylcholine receptor (nAChR) antagonist, hexamethonium. Isolated perfused rat hearts were randomly assigned to one of the following 4 groups: (1) sham IPC, hearts received a 40-min stabilisation period; (2) control + hexamethonium (50 μM), hearts received a 10-min stabilisation period followed by 35-min perfusion with 50 μM hexamethonium. (3) IPC3, hearts received three cycles of 5-min global ischaemia with intermittent 5-min reperfusion immediately prior to index ischaemia; (4) IPC3 + hexamethonium (50μM), same as group 4, however, the hearts were treated with hexamethonium for 5 min prior to and the duration of the 3-cycle preconditioning.

The second experiment examined the importance of muscarinic acetylcholine receptors (mAChR) in classical IPC, via use of the drug atropine. Hearts were randomly assigned to one of 3 groups: (1) control + atropine (100 nM), hearts received a 10-min stabilisation followed by 35-min perfusion with 100 nM atropine; (2) IPC3, hearts received three cycles of 5-min global ischaemia with intermittent 5-min reperfusion immediately prior to index ischaemia; (3) IPC3 + atropine (100 nM), same as group 2, however, hearts were perfused with atropine for 5 min prior to and the duration of the IPC protocol.

All hearts subsequently received 35-min LAD ischaemia and 60-min reperfusion. At the end of the protocol, hearts were analysed for infarct size using methods described below.

## Study 2

### Classical IPC with coronary effluent transfer to naïve isolated hearts

This study uses a model first pioneered by the Przyklenk laboratory [17], and has been used in several subsequent publications by different groups [11, 43]. Although it is described in the literature as more similar to remote ischaemic preconditioning, in fact it likely reflects the humoral aspect to classical preconditioning. That is, it enables one to investigate the factors released by the heart following IPC. RIC is now generally agreed to occur via a more complex neuro-humoral pathway [55].

In the first part of the experiment, coronary effluent was collected from isolated perfused donor rat hearts, randomised into one of the following two groups: (1) donor control hearts underwent 30 min of perfusion during which effluent was collected; (2) donor IPC hearts received three cycles of 5-min global ischaemia with intermittent 5-min reperfusion, during which effluent was collected.

In the second part, recipient hearts were perfused with effluent (from above) for 10 min, following 30 min of stabilisation, immediately prior to 35-min LAD ischaemia and 60-min reperfusion. These recipient hearts were randomised to one of four groups: (1) *C*_eff_, hearts received 10-min perfusion of donor control effluent immediately prior to ischaemia; (2) IPC_eff_, hearts received a matched 10-min perfusion of donor IPC effluent; (3) *C*_eff_ + Hex, the same as group 1, however, hearts were perfused with hexamethonium (50 μM) for 5 min prior to and the duration of effluent perfusion; (4) IPC_eff_ + Hex, same as group 2, however, hearts were perfused with hexamethonium for 5 min prior to and the duration of effluent perfusion. Following reperfusion, all hearts were analysed for infarct size using methods described below.

### Acetylcholine assay

A Choline/Acetylcholine Assay Kit (Abcam, UK) was used to measure the concentration of acetylcholine in effluent collected following IPC (3 × 5-min global ischaemia–reperfusion) or corresponding control period, as described above. The assay was carried out in accordance with the instructions provided by the manufacturer. Briefly, via the use of acetylcholinesterase, the level of free and total choline was measured in each sample, enabling an estimation of the concentration of ACh within the sample.

### Classical IPC with coronary effluent transfer to naïve isolated cardiomyocytes

In order to ascertain the role of the intrinsic cardiac nerves in classical IPC we undertook a series of studies using the isolated cardiomyocyte, where nerves are not present. Isolation of adult male Sprague–Dawley rat (250–300 g) cardiomyocytes was performed using a previously described protocol [30]. Cells were plated on laminin-coated 35-mm dishes (VWR international, PA, USA) and left to stabilise for 24 h prior to use. Dishes were assigned to one of the following groups: (1) normoxia, cells were left in M119 media for the duration of the protocol; (2) vector control, cells were stimulated for 10 min with Krebs–Henseleit buffer; (3) *C*_eff_, cells were stimulated for 10 min with control effluent; (4) IPC_eff_, cells were stimulated for 10 min with IPC effluent; (5) NECA, cells were stimulated with the adenosine A2B receptor agonist, 5′-*N*-ethylcarboxamidoadenosine (NECA). Following stimulation (groups 2–5), cells were treated with hypoxic buffer (NaCl 127.8 mM, 14.8 mM KCl, KH_2_PO_4_ 1.2 mM, MgSO_4_ 1.2 mM, NaHCO_3_ 2.2 mM, CaCl_2_ 1 mM, Na. lactate 10 mM, gassed with 5% CO_2_–95% N_2_ to achieve pH 6.4), and placed into a sealed hypoxic chamber (Billups-Rothenberg, CA, USA) filled with 5% CO_2_–95% N_2_ gas mix. Hypoxia was continued for 3 h at 37 °C, at which point the cells were removed from the chamber and treated with normoxic buffer (glucose 10 mM, NaCl 118 mM, KCl 2.6 mM, KH_2_PO_4_ 1.2 mM, MgSO_4_ 1.2 mM, NaHCO_3_ 22 mM, CaCl_2_ 1 mM, gassed with 5% CO_2_–95% O_2_ to achieve pH 7.4) to simulate reperfusion. The reoxygenation was continued for 1 h, at which point the proportion of cell death was measured via propidium iodide staining and confocal microscopy (previously described here [65]).

### Infarct size assessment

Infarct size of each isolated heart in the above experiments (Study 1 and 2) was calculated using the following methods, described in detail previously [9]. Briefly, at the end of the reperfusion period, the LAD suture was re-tightened and 1 ml of 0.25% Evans blue dye was perfused through the heart in order to delineate the area-at-risk of infarction. The hearts were then frozen at −20°C before being sectioned into 5 transverse slices and stained for viable tissue by immersion in 1% triphenyl-tetrazolium chloride at 37°C for 15 min. Following fixation in 10% formalin for 24 h, the sections were digitally scanned to a computer for analysis. Analysis of infarct size (IS) as a proportion of area at risk (AAR) was calculated via planimetry using imageJ software (version 1.45, National Institutes of Health, USA).

### Statistical analysis

Data groups were first analysed for normality using the Kolmogorov–Smirnov test. Statistical differences between two groups were analysed using a Student’s *t* test and more than two groups using a one-way analysis of variance (ANOVA) with Tukey’s multiple comparison post-test. All data are presented as mean ± standard error of the mean (SEM). Data groups were classed as significantly different with a *p* value less than 0.05. Notation of significance is described in figure legends. Analyses were performed using GraphPad Prism version 5 for Windows (CA, USA).

## Results

### Study 1: classical ischaemic preconditioning is abrogated by hexamethonium and atropine

**Fig. 2:**
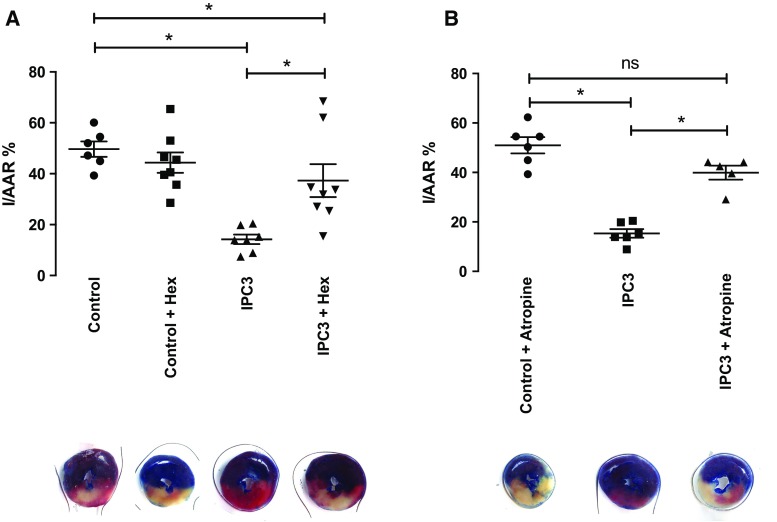
Hexamethonium and atropine abrogate ischaemic preconditioning: **a** Hexamethonium abrogates preconditioning induced by both one and three cycles of IPC (*n* = 6–8 per group, *asterisk* = *p* < 0.05 vs control); **b** atropine also abrogates the protection afforded by IPC3 (*n* = 6 per group except IPC + atropine where *n* = 5, *asterisk* = *p* < 0.05 vs control + atropine). Data presented as mean ± SEM

In our isolated perfused rat heart model we demonstrated that three cycles of IPC was effective at reducing infarct size relative to control (IS/AAR = 14 ± 2% vs control IS/AAR = 48 ± 3%, *p* < 0.05) (Fig. 2a). The nAChR antagonist, hexamethonium (50 μM), almost fully abrogated this cardioprotection (IS/AAR = 37 ± 7%, *p* > 0.05 vs control). Hexamenthonium alone did not influence infarct size (IS/AAR = 44 ± 4%).

In the second part of this study, the muscarinic antagonist, atropine, was used to investigate the pathway downstream of intrinsic ganglia. 100 nM atropine did not affect infarct size (IS/AAR = 51 ± 3%); however, it abrogated the cardioprotection induced via three cycles of IPC (IS/AAR = 40 ± 3% vs IPC = 15 ± 2%) (Fig. 2b).

### Study 2: factors released following classical IPC require intrinsic cardiac nerves to induce protection

**Fig. 3:**
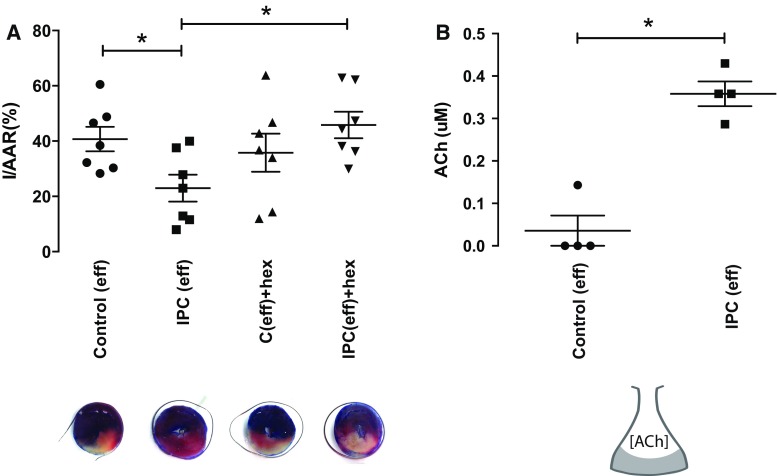
IPC effluent protects the naïve isolated heart from ischaemia–reperfusion injury. **a** Effluent collected following IPC significantly protected a naïve isolated rat heart via a hexamethonium-sensitive mechanism (*n* = 7–8 per group, *asterisk* = *p* < 0.05 vs control effluent); **c** the concentration of ACh in coronary effluent increases tenfold following IPC (*n* = 4 per group, *asterisk* = *p* < 0.05 vs Control effluent). Data presented as mean ± SEM

Effluent collected from hearts following classical IPC induced significant protection when perfused through a second or naïve isolated rat heart prior to acute IRI (IS/ARR = 19 ± 2, *p* < 0.05 vs control IS/AAR = 46 ± 6%). Pre-treatment of the naïve recipient heart with the nicotinic antagonist, hexamethonium, abrogated the protection offered by IPC effluent (IS/AAR = 46 ± 5%, *p* < 0.05 vs IPC_eff_) (Fig. 3a).

A large release of ACh following IPC was observed in these isolated perfused rat hearts, with a tenfold increase in the concentration relative to control effluent (IPC_eff_ = 0.36 ± 0.03 μM vs *C*_eff_ = 0.04 ± 0.04 μM, *n* = 4, *p* < 0.001) (Fig. 3b). Three of the four control effluent samples did not contain any detectable ACh.

### Classical IPC effluent appears not to protect isolated cardiomyocytes from simulated IRI

**Fig. 4:**
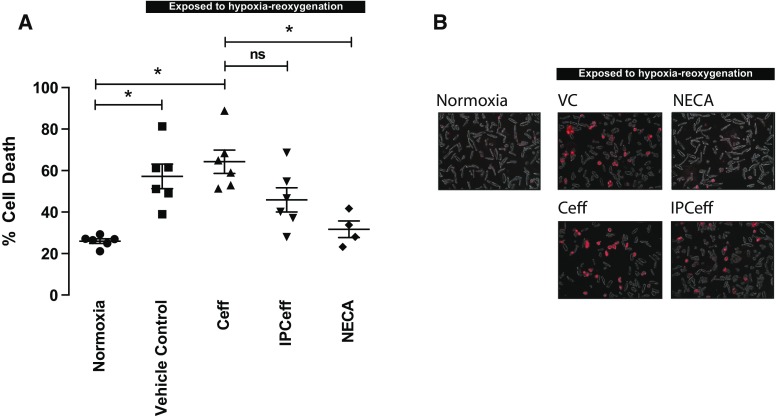
IPC effluent does not protect isolated cardiomyocytes from simulated ischaemia–reperfusion injury. **a** Isolated rat cardiomyocytes were not protected from hypoxia-reoxygenation injury by prior exposure to IPC effluent. The adenosine A2B agonist, NECA, was able to reduce cell death significantly (*n* = 6 in all groups except *n* = 4 for NECA, *asterisk* = *p* < 0.05 vs normoxia); **b** representative images of isolated cardiomyocytes subjected to the different protocols, the *red staining* indicate dead cells. Data presented as mean ± SEM

Cells that were maintained under normoxic conditions throughout the experiment exhibited 27 ± 2% cell death (Fig. 4). In cells that underwent simulated IR, this was increased to 57 ± 6 and 64 ± 6% after pre-treatment with the vehicle control and control effluent, respectively (*p* < 0.001 vs normoxic in both cases). Treating the cells with IPC effluent did not significantly reduce cell death (45 ± 6%, *p* = 0.09 vs *C*_eff_). The adenosine A2b agonist NECA (used as a +ve control) significantly reduced cell death to 32 ± 4% (*p* < 0.01 vs vector control and *C*_eff_) (Fig. 4).

## Discussion

These results are the first indication of a neural pathway in the mechanism of classical ischaemic preconditioning. We demonstrated an important role for nicotinic and acetylcholine receptors, suggesting that intrinsic cardiac ganglia remain active in the isolated heart preparation and are important in conveying the protective message. Acetylcholine is released from the heart following IPC, perhaps from parasympathetic post-ganglionic nerve endings in the ventricles, and induces protection via an atropine-sensitive mechanism. The lack of total abolition of protection, in the presence of ganglionic or muscarinic antagonism, is likely due to the fact that several factors contribute to the IPC mechanism in the isolated heart model [18]. Moreover, coronary effluent collected following IPC was able to protect a naïve isolated heart, but not isolated cardiomyocytes, from IRI. The protection in naïve hearts was abrogated by hexamethonium, highlighting the importance of intrinsic cardiac ganglia in the mechanism of IPC. We therefore propose that IPC is governed, in part, via a neuro-humoral pathway; a factor released following IPC activates intrinsic cardiac ganglia, leading to release of ACh from parasympathetic post-ganglionic nerve endings in the ventricles, thus inducing cardioprotection via activation of muscarinic receptors. These data are, to some extent, additive to our previous study, where we proved an important role for intrinsic cardiac ganglia in remote ischaemic conditioning. Whilst a neural pathway has been well validated in RIC, this is the first to imply a similar intrinsic cardiac neural pathway in IPC. Thus, there appear to be more similarities between the mechanisms of classical (direct) and remote ischaemic conditioning than were previously apparent [5, 55].

### Intrinsic cardiac ganglia in the isolated heart

Intrinsic cardiac ganglia are widely distributed in the myocardium and not only relay central efferent pre-ganglionic information, but also are able to process sensory afferent information from the myocardium and control efferent post-ganglionic firing [7, 46]. Moreover, several anatomical and functional studies have indicated a significant presence of vagal neurons in the ventricles, in addition to sympathetic and local circuit neurons [3]. The intrinsic cardiac nervous system is therefore able to control cardiac indices on a beat-to-beat basis, in the absence of input from the central nervous system [4].

Activation of sensory afferent nerves in the Langendorff heart has previously been demonstrated to induce cardioprotection. Perfusion with capsaicin, a known activator of C-fibre afferents, induced early and delayed protection against ischaemia–reperfusion injury in isolated Langendorff rat hearts [69]. In our study, ganglionic antagonism abrogated the protection afforded by IPC, suggesting sensory afferent activation occurs following IPC and is important in conferring the cardioprotection. We further demonstrated a tenfold increase in the ACh concentration in perfusate collected following IPC. This suggests activation of post-ganglionic parasympathetic neurones from the intrinsic ganglia. Indeed, muscarinic antagonism, using atropine, abolished IPC-induced cardioprotection. The parasympathetic nervous system has a well-defined cardioprotective effect [34, 48, 49, 62], and recently emerged as a key mediator of the cardioprotection afforded by “remote” ischaemic conditioning (RIC) [6, 49, 55]. Indeed, a recent study demonstrated that increased parasympathetic tone ameliorated the functional and structural remodelling of the intrinsic cardiac nervous system following myocardial infarction [8]. Thus, we propose that there exist similarities between the mechanism of remote and classical ischaemic conditioning [37, 54]; an intrinsic neural reflex loop in response to the brief ischaemia of IPC, which activates cardiac ganglia and increases post-ganglionic vagal tone, leading to release of ACh in the ventricles.

### A contentious role for acetylcholine in IPC

IPC is triggered via release of several small molecules, and their subsequent receptor activation in the myocardium. This was first demonstrated by Liu et al., who showed that pre-treatment with an adenosine receptor antagonist abrogated IPC in rabbit hearts [45]. Moreover, perfusion of exogenous adenosine through the heart prior to infarction mimicked the cardioprotection of IPC. These data suggested endogenous release of adenosine occurs in response to IPC, which protects the myocardium. Two other small molecules, opioids [14, 47, 60] and bradykinin [66], were found in subsequent studies to be important in the mechanism of IPC, via activation of their receptor. These appear to be connected via a common intracellular cytoprotective signalling pathway [53], which centres on activation of protein kinase C (PKC) [18]. Coronary effluent collected following IPC can protect a naïve isolated heart from infarction [11, 16], supporting the theory of a humoral trigger for IPC. Indeed, adenosine is released into the effluent following IPC, and confers protection to the naïve heart via crosstalk with opioid receptors [15, 43]. Bradykinin is not involved in this setting due to the requirement for circulating kininogens in the blood, not present in the isolated buffer-perfused model [21].

The role for acetylcholine in IPC, however, is more contentious. While several studies from Krieg et al. demonstrated exogenous ACh could induce cardioprotection in the Langendorff model, the same group discounts its involvement in the mechanism of IPC [38–40]. However, two studies from Kawada et al. demonstrated brief, 5-min ischaemia in in vivo rabbit and cat models induces interstitial release of ACh in the ventricles [35, 36]. Data from our study confirm those of Kawada et al. with a significant release of ACh observed in coronary effluent following IPC. Given this observation, we hypothesised that isolated cardiomyocytes would be protected from simulated IRI following exposure to coronary effluent. Presumably, if ACh was mediating protection here, it would act directly on muscarinic receptors on the cardiomyocytes in the naïve heart. However, IPC effluent was not able to significantly reduce cell death in isolated cardiomyocytes subjected to hypoxia-reoxygenation. A small reduction in cell death is observed, likely due to the presence of several trigger factors in the effluent [15, 43]; however, this was not statistically significant. This is perhaps due to dilution of factors released from the myocardium in the effluent following IPC, such that the concentration in the isolated cells would be insufficient for cardioprotection. However, we did not investigate whether exogenous ACh of the same concentration could induce cardioprotection in isolated cardiomyocytes or Langendorff models.

Finally, coronary effluent collected following IPC induced powerful cardioprotection when perfused through naïve isolated rat hearts, via a mechanism also sensitive to hexamethonium. This experiment confirms the key point to the study; factors released following IPC require intrinsic cardiac ganglia to induce cardioprotection. The neural and humoral components to IPC, therefore, are co-dependent.

### Is there a common trigger for classical and remote ischaemic conditioning?

Our data suggest that both classical and remote ischaemic conditioning (RIC) may share a common trigger pathway; i.e. local release of an autocoid, activation of sensory afferent nerves and subsequent recruitment of the intrinsic cardiac nervous system. RIC is induced via the same principle as classical IPC, brief cycles of ischaemia, however applied to a region remote from the heart [10]. The trigger for RIC is thought to be local release of an autocoid, such as adenosine, which activates sensory afferent nerves communicating the protective message away from the conditioned limb. Indeed, a small injection of adenosine into the femoral artery is sufficient to induce cardioprotection [64], as is activation of C-fibre sensory afferents by capsaicin [58] or transcutaneous electrical nerve stimulation [50]. Moreover, our study has demonstrated the importance of intrinsic cardiac ganglia in IPC, which necessitates prior sensory nerve activation in the heart. Classical IPC is known to involve release of adenosine and calcitonin gene-related peptide [43, 45], both of which are know to activate sensory afferent nerves. Perhaps, therefore, these are the trigger for this aspect to IPC. The trigger for both RIC and classical IPC appear to share important similarities, with both neural and humoral components [55].

A recent meta-analysis revealed that ischaemic preconditioning had variable efficacy in mammalian species; namely, IPC was more effective in rodents relative to non-rodents [67]. Myocardial autonomic innervation is known differ according to the species’ size [59]; however, it is not clear whether these differences relate to the species-specific effect of IPC. Finally, with respect to remote ischaemic conditioning, although there appear to be differences in the signalling cascades important for cardioprotection between species [63], a recent meta-analysis revealed no difference in the efficacy of cardioprotection relative to species [12].

Finally, it is important to note the range of different factors that contribute to the trigger phase of IPC, in addition to the release of autacoids and neuro-humoral factors. Physical stimuli, such as activation of stretch receptors during the preconditioning stimulus, have been demonstrated to reduce infarct size [24]. In addition, reactive hyperaemia during brief reperfusion induces release of nitric oxide from endothelial cells, which can trigger IPC, although this effect appears to be species specific [52, 56]. Several other intracellular chemical stimuli have been implicated in the trigger phase of IPC. The brief ischaemia and reperfusion of IPC induces release of reactive oxygen species in cardiomyocytes, which in small quantities can induce cardioprotection [31]. Thus, IPC is triggered via a variety of stimuli, all of which contribute to reaching the appropriate threshold for cardioprotection to ensue.

### Study limitations

The key limitation of the study lies in the specificity of hexamethonium to neuronal nicotinic acetylcholine receptors. Hexamethonium is widely used within the literature as a ganglionic blocker, being a neuronal nicotinic acetylcholine receptor antagonist. Although it has some affinity to muscarinic M2 receptors, this becomes negligible at concentrations below 100 µM [20], and it has an IC_50_ of 11 µM at nAChRs [23]. Thus, for the purposes of this study 50 µM was used in order to achieve high specificity to nicotinic receptors, but the potential may remain for non-specific effects. The second issue relates to the spatial expression of nicotinic receptors within the myocardium. Although nicotinic receptors are largely limited to the intrinsic cardiac ganglia, there is evidence for expression of the receptors on the myocytes [19]. Thus, there is a small possibility that hexamethonium is exerting its effect through non-ganglionic action.

Secondly, these data do not fully ascertain the role of ACh within the effluent. An important additive experiment would be to treat a naïve isolated heart with acetylcholine at the same level observed within the effluent. In addition, it is possible that stimulation of isolated cardiomyocytes with effluent for longer than 10 min could reveal cardioprotection.

## Conclusion

This is the first study to implicate intrinsic cardiac ganglia in the mechanism of classical ischaemic conditioning. We propose that IPC activates an intrinsic cardiac neural reflex and is an important part of the cardioprotective mechanism. This is a significant finding for several reasons. The Langendorff perfused heart was traditionally thought of as a denervated preparation; however, clearly this is not true given the current data, in addition to our recent publication [55]. Moreover, these data add to the paradigm that IPC is a receptor-mediated phenomenon. However, there seems to be an added layer of complexity, with the intrinsic ganglia responsible for conferring a portion of the cardioprotection. This is of importance given the issue of co-morbidities in the clinical setting. For example, diabetes has been well documented to decrease the efficacy of IPC [68]; perhaps this could be explained by the peripheral sensory neuropathy that occurs as the disease progresses [33]. Whether the peripheral sensory nerve activation induced via remote ischemic conditioning is comparable to that of classical ischaemic conditioning is not clear. Further work is necessary to ascertain the exact nature of the involvement of intrinsic ganglia in this setting.

## Abbreviations

AAR: Area-at-risk
ACh: Acetylcholine
AMI: Acute myocardial infarction
CFR: Coronary flow rate
CNS: Central nervous system
IPC: Ischaemic preconditioning
IRI: Ischaemia–reperfusion injury
IS: Infarct size
LAD: Left anterior descending
LVEDP: Left ventricular end-diastolic pressure
mAChR: Muscarinic acetylcholine receptor
MI: Myocardial infarction
nAChR: Nicotinic acetylcholine receptor
TTC: Triphenyl tetrazolium chloride
NECA: 5′-*N*-Ethylcarboxamidoadenosine
